# The Temporal Prediction of Stress in Speech and Its Relation to Musical Beat Perception

**DOI:** 10.3389/fpsyg.2018.00431

**Published:** 2018-04-03

**Authors:** Eleonora J. Beier, Fernanda Ferreira

**Affiliations:** Department of Psychology, University of California, Davis, Davis, CA, United States

**Keywords:** rhythm, stress, speech, music, meter, prediction, prosody, language

## Abstract

While rhythmic expectancies are thought to be at the base of beat perception in music, the extent to which stress patterns in speech are similarly represented and predicted during on-line language comprehension is debated. The temporal prediction of stress may be advantageous to speech processing, as stress patterns aid segmentation and mark new information in utterances. However, while linguistic stress patterns may be organized into hierarchical metrical structures similarly to musical meter, they do not typically present the same degree of periodicity. We review the theoretical background for the idea that stress patterns are predicted and address the following questions: First, what is the evidence that listeners can predict the temporal location of stress based on preceding rhythm? If they can, is it thanks to neural entrainment mechanisms similar to those utilized for musical beat perception? And lastly, what linguistic factors other than rhythm may account for the prediction of stress in natural speech? We conclude that while expectancies based on the periodic presentation of stresses are at play in some of the current literature, other processes are likely to affect the prediction of stress in more naturalistic, less isochronous speech. Specifically, aspects of prosody other than amplitude changes (e.g., intonation) as well as lexical, syntactic and information structural constraints on the realization of stress may all contribute to the probabilistic expectation of stress in speech.

## Introduction

In the domain of music, it is well established that metric structure gives rise to expectancies which allow humans to perceive and synchronize to a beat (Large and Kolen, 1994; Large and Jones, 1999). As music and language share many cognitive mechanisms and neural resources (Patel, 2008), the same beat perception mechanisms applied to musical rhythm might also be used when processing speech rhythms, involving the representation of a metrical structure and prediction of where the next stressed syllable will occur. Rhythmic properties of speech based on the alternation of stressed and unstressed syllables have been posited to be organized into hierarchical metrical trees or grids, which may be similar to musical meter (e.g., Martin, 1972; Liberman and Prince, 1977; Selkirk, 1984; Ferreira, 1993, 2007). However, while prediction mechanisms are thought to underlie many aspects of language processing (Federmeier and Kutas, 1999; Pickering and Garrod, 2007; Kuperberg and Jaeger, 2016), it is less clear to what extent rhythmic features of speech are predicted during on-line comprehension.

While rhythmic perception in music operates on periodic, hierarchically organized beats, the same periodicity is seldom found in naturalistic speech (Lehiste, 1977; Dauer, 1983). Thus, while still presenting rhythmic properties, the stress patterns of speech may typically be too varied to give rise to meaningful expectations (Patel, 2008; London, 2012). On the other hand, there are several reasons why predicting when the next stressed syllable will occur in speech may be useful. Stress patterns are an important segmentation cue in speech and in language acquisition (Sanders and Neville, 2000; Nazzi and Ramus, 2003), and aid word recognition in the presence of many competing lexical items (Norris et al., 1995). Thus, successful prediction of the occurrence of stresses may aid the difficult task of breaking the continuous speech signal into its components.

The temporal prediction of stress could also be beneficial by reducing processing costs during language comprehension. Stressed syllables are detected and processed faster than unstressed ones (Cutler and Foss, 1977; Gow and Gordon, 1993). Crucially, shorter reaction times (RTs) are also found when an acoustically identical phoneme is predicted to be stressed based on various aspects of the preceding context (Cutler, 1976; Cutler and Fodor, 1979; Pitt and Samuel, 1990). Stressed syllables are thought to carry higher informational content than unstressed ones (Altman and Carter, 1989), and stress patterns appear to be strongly related to the information structure of a sentence (Aylett and Turk, 2004; Calhoun, 2010). Therefore, in order to more easily integrate new information, listeners may predict the timing of future stresses and allocate attention and processing resources to those points in time. This has been termed the Attentional Bounce Hypothesis (ABH) (Pitt and Samuel, 1990). The theoretical background surrounding this hypothesis is discussed below. We then turn to evidence for and against temporal prediction of stress in speech, its possible neural mechanisms, and its limitations.

## How Are Predictions Formed?

Most work on the prediction of stress in speech assumes that listeners form expectations based on perceived regularities in the stress pattern of a sentence. Originally, through the definition of English as a “stress-timed” language, regularity was thought to consist of the physical periodicity of stresses occurring at close to isochronous intervals (Abercrombie, 1967), which would easily lead a listener to infer the next occurrence of a stress. However, naturalistic speech does not present this degree of periodicity (Lehiste, 1977). While the periodicity of stress may be primarily a perceptual phenomenon (Lehiste, 1977), as captured in the notion of perceptual centers (p-centers; Morton et al., 1976), this claim is controversial, and there is no consensus regarding the presence of isochrony either in the signal or as a perceptual experience.

Regularities in speech stress patterns have also been characterized through hierarchical metrical trees or grids. This concept comes from phonological theories designed to explain how stress is distributed in a sentence (e.g., Martin, 1972; Liberman and Prince, 1977; Hayes, 1983; Selkirk, 1984). A basic tenet of most theories is the avoidance of stress clashes or lapses (two stressed or two unstressed syllables next to each other; e.g., thirTEEN turns into THIRteen MEN), which in practice renders the pattern of stresses more periodic (Selkirk, 1984). However, these theories focus primarily on the hierarchical nature of stress structure and not on periodicity (Martin, 1972). The timing and prominence of every event in a sequence is determined by that of all other sounds through an internal, hierarchical structure, in opposition to sounds concatenated at a single level, as in the case of a simple isochronous beat.

Martin (1972) proposed that listeners internalize this hierarchical structure during on-line comprehension and can thus predict the location of future stresses. This in turn would allow them to allocate their attention to those points in time, facilitating processing, a concept that was later termed the ABH (Pitt and Samuel, 1990). However, as we will argue, research following this proposal focused less on hierarchical stress structure and more on expectancies based on periodicity. While Martin frames these as very different types of predictions, the two are not always distinguished in the literature.

We believe much of this confusion arises from an inconsistency in the way terms such as “rhythm” and “meter” are defined. For the sake of clarity we adopt the following definitions, though we acknowledge they are not necessarily the only or best ways to interpret these terms. We view rhythm as an informal way to refer to temporal patterning of events, whereas we treat meter as a specific type of structure. Based on Patel (2011), we define meter as a “hierarchical organization of beats in which some beats are perceived as stronger than others” (Patel, 2011, 100), where beats may be constituted by the accents or stresses found in both music and language. Importantly, this definition highlights the hierarchical nature of metric structure and it is thus more in line with theories of metrical grids described above. While this type of structure may tend toward periodicity, such as through stress shift, its realization need not necessarily be periodic.

## Evidence

Several early studies of the prediction of stress in speech utilized phoneme monitoring as an indication of processing speed (e.g., Shields et al., 1974; Cutler, 1976; Pitt and Samuel, 1990; Quené and Port, 2005). Shields et al. (1974) found shorter RTs to phonemes belonging to stressed syllables of nonsense words as opposed to when the same syllables were unstressed. The nonsense words were embedded in sentences, based on the idea that a sentence’s stress pattern induces timing expectancies for future stresses (Martin, 1972). However, this experiment did not entirely rule out the possibility that the acoustic saliency of stressed syllables, rather than their temporal predictability, may have facilitated processing (Cutler, 1976; Pitt and Samuel, 1990). Such acoustic differences were controlled for in a subsequent test of the ABH (Pitt and Samuel, 1990, Exp. 1). Two-syllable words which could be accented on the first or second syllable (verb-noun pairs such as PERmit vs. perMIT) were embedded within sentences. Acoustic differences were controlled by creating a single “neutral stress” version of the words (PERMIT). The authors expected RTs to be shorter when the target phoneme occurred on a syllable that had been predicted to be stressed based on the preceding rhythmic context. However, this was not the case, suggesting that the difference in RTs found by Shields et al. (1974) might in fact have been due to the stressed syllables’ acoustic saliency. Nonetheless, while both Shields et al. (1974) and Pitt and Samuel (1990) based their hypotheses on theories of metrical grids, the meter of the sentences was not itself controlled for; additionally, factors other than stress rhythm may have confounded the effects of timing expectancies (e.g., semantic and syntactic prediction for whether a verb or a noun would occur in Pitt and Samuel, 1990). Thus it is hard to tell from these early studies whether rhythmic predictions are indeed at play in speech.

Subsequent studies induced temporal expectations for stress through the periodic or semi-periodic alternation of stressed and unstressed syllables (e.g., Pitt and Samuel, 1990, Exp. 2; Quené and Port, 2005; Schmidt-Kassow and Kotz, 2009a,b; Rothermich et al., 2012; Rothermich and Kotz, 2013). In their second experiment, Pitt and Samuel (1990) embedded neutral stress targets in strings of bisyllabic words presenting the same or opposite stress pattern as the target – either trochaic (S-w) or iambic (w-S). While in this case RTs were shorter for syllables predicted to be stressed based on the preceding rhythm, in their discussion Pitt and Samuel (1990) cast doubt on whether this result would generalize to natural sentences. One study using a similar methodology found that precise timing regularity (isochrony of stresses), rather than consistency in the metric pattern of the target and the preceding words, best explains differences in RTs (Quené and Port, 2005). This speaks against the claim that stress periodicity is primarily a perceptual phenomenon, suggesting that rhythmic predictions for stress are most reliably induced by physical periodicity of the stimulus – which is seldom found in natural speech (though see Otterbein et al., 2012).

Nonetheless, recent studies provide evidence for rhythmic expectancies induced by sentences comprised of bisyllabic words with consistent trochaic or iambic patterns, but lacking exact isochrony. Schmidt-Kassow and Kotz (2009b) observed event-related brain potentials to “metric violations” induced by having a target word be pronounced with incorrect lexical stress (e.g., with a trochaic pattern rather than the correct iambic pattern) within such sentences. A biphasic pattern was observed, consisting of an early anterior negativity and a P600 effect. Schmidt-Kassow and Kotz (2009a) found similar results when using correctly pronounced target words with a stress pattern opposite to the preceding context (although this effect was only present when listeners were instructed to actively pay attention to the meter of the sentences). Additionally, Rothermich et al. (2012) observed a reduced N400 to semantically unpredictable target words when the words were embedded in sentences with regular (iambic or trochaic) rather than irregular stress patterns. This suggests that temporal regularity of stress in speech leads to expectations which in turn may facilitate semantic integration of unpredicted words. Lastly, eye-tracking evidence for the prediction of lexical stress was found for stimuli with a highly constraining metrical structure (limericks; Breen and Clifton, 2011). These experiments corroborate evidence from phoneme monitoring, suggesting that the alternation of stressed and unstressed syllables may contribute to timing expectancies for stress, though they do not speak to whether such predictions may be at play in naturally occurring speech.

## Entrainment

The ABH (Shields et al., 1974; Pitt and Samuel, 1990) suggests that attention is directed to the predicted location of a stress regardless of how this location is predicted. Later studies, inducing rhythmic expectancies through periodicity, shifted their theoretical approach to the more specific notion of neural entrainment, as assumed in Dynamic Attending Theory (DAT; Large and Kolen, 1994; Large and Jones, 1999). This theory, which was primarily developed to understand the perception of musical rhythm, posits that listeners form expectations for when a beat will occur thanks to the entrainment, or synchronization, of their own neural oscillations with an external periodic stimulus. This leads to the dynamic allocation of attention to specific points in time. Neural oscillations have been shown to entrain to rhythmically organized stimuli (Lakatos et al., 2008), and to the strong beats of an imagined meter imposed over a periodic series of acoustically equal beats (Nozaradan et al., 2011).

While many have posited that this mechanism may also be involved in the perception of stress patterns in speech (e.g., Large and Jones, 1999; Port, 2003; Ghitza and Greenberg, 2009; Kotz and Schwartze, 2010; Goswami, 2012; Peelle and Davis, 2012), whether this is the case has not been unequivocally established. Cummins and Port (1998) found that in a speech cycling task (where a phrase was repeated multiple times in synchrony with a metronome), stresses tended to align with particular metrical positions in accordance with the principles of DAT (Port, 2003). However, it is not clear whether this work applies to regular speech and whether it translates to neural activity.

A promising lead comes from work positing a role for entrainment in the segmentation and temporal prediction of speech units at different timescales (for a review, see: Kösem and van Wassenhove, 2017; Meyer, 2017). Neural oscillations in the theta range (4–8 Hz) have been shown to synchronize with fluctuations in the temporal envelope of speech corresponding to the syllabic rate (Giraud and Poeppel, 2012; Peelle and Davis, 2012). Entrainment to the syllabic rate may be a fundamental mechanism for prediction, segmentation, and speech processing in general (Ghitza and Greenberg, 2009; Peelle and Davis, 2012). Oscillations in the delta range (0.5–4 Hz) have also been found to track the pitch contour of speech, possibly reflecting entrainment to intonational boundaries (Bourguignon et al., 2013). Entrainment is posited to be a fundamental element of neural mechanisms supporting the representation of temporal structure and temporal predictions in speech (Kotz and Schwartze, 2010; Schwartze and Kotz, 2013). To the best of our knowledge, however, neural entrainment to hierarchically organized stress patterns in speech remains to be empirically established.

## Prediction in Everyday Speech

The evidence for temporal prediction of stress reviewed thus far has relied on stimuli that contain a certain degree of periodicity, induced either through perfect isochrony (Quené and Port, 2005) or through the regular alternation of stressed and unstressed syllables (Pitt and Samuel, 1990; Schmidt-Kassow and Kotz, 2009a,b; Rothermich et al., 2012). The neural mechanism most commonly associated with these findings is the entrainment of neural oscillations to an external stimulus, in this case the pattern of stresses in speech. While entrainment may not require exact isochrony and can adjust to various types of rhythmic irregularity (Large and Jones, 1999; Large et al., 2002), it is not clear whether the stress patterns of natural speech present the coherence required by neural oscillations to entrain (Schwartze and Kotz, 2013). Schwartze and Kotz (2013) note that different types of speech may present more or less rhythmic regularity, and may therefore engage entrainment to various degrees.

Whether everyday speech presents enough rhythmic regularity to induce temporal expectations for stress through neural entrainment remains an open question. It is likely that neural entrainment is at play in the presently reviewed literature, as these studies induced expectancies through the periodic (or semi-periodic) presentation of stress. However, their generalization to the perception of natural speech remains to be tested. Given that natural speech presents far less periodicity than the stimuli utilized in these studies, two possibilities arise: (1) the prediction of stress in natural speech is reduced or non-existent, (2) mechanisms other than neural entrainment are involved in the prediction of stress. These two possibilities and their implications are explored in the following sections.

### No Prediction?

It is possible that the prediction of stress observed in the studies here reviewed relies on general beat perception processes not typically utilized in speech perception. In other words, listeners may indeed entrain to the beat induced by the periodic presentation of stressed syllables, but the same phenomenon could have been induced by any other periodic stimulus (such as a simple sequence of beats, as in Cason and Schön, 2012). These predictions may therefore be tied to the specific manipulations of these experiments and not be present in everyday speech perception. This reflects the observation that the role of prediction mechanisms in language comprehension may have been overestimated through the use of overly predictable stimuli (Huettig and Mani, 2016).

However, it is unlikely that no prediction of stress is involved in natural speech. In the absence of prediction, any difference in RTs to stressed as opposed to unstressed syllables would be due to bottom-up, salient features of stressed syllables. This, however, is inconsistent with the finding that shorter RTs are shown even for syllables predicted to be stressed in absence of acoustic differences (Cutler, 1976; Cutler and Fodor, 1979). Additionally, it is not clear why phenomena such as stress shift would constrain the stress patterns of speech into hierarchically organized structures allowing prediction. Finally, the hierarchical nature of stress patterns suggests that prediction for the location of the strongest stress in a sentence (i.e., the nuclear stress) is required in order not to misclassify a pre-nuclear (relatively weaker) stress as nuclear (Calhoun, 2010). Thus, it is likely that mechanisms other than neural entrainment are involved in the prediction of stress for everyday speech.

### Other Forms of Prediction

While the majority of studies have induced temporal predictions of stress by controlling the periodicity of the preceding speech, a few experiments have achieved similar results through different manipulations. Prosodically, the prediction of stress has been induced through a sentence’s intonational contour (Cutler, 1976), as well as through the manipulation of the duration and the pitch quality of vowels preceding a target word (Brown et al., 2016).

This points to the idea that stress patterns may be better conceptualized in conjunction with other prosodic features such as intonation, rather than solely through fluctuations in amplitude envelope (e.g., as in Peelle and Davis, 2012). Special qualities in amplitude, pitch, duration, and spectral tilt are all thought to contribute to the perception of stress (Fry, 1955; Shattuck-Hufnagel and Turk, 1996; Sluijter and van Heuven, 1996; Breen et al., 2010). As context-dependent predictions have been observed for prosodic elements such as pitch accents (Weber et al., 2006; Dimitrova et al., 2012), prosodic boundaries (Clifton et al., 2002), and prominence shifts (Klassen and Wagner, 2017), these elements may need to be modulated as well, or at least controlled for, in order to fully understand the prediction of stress.

Moreover, the acoustic realization of a sentence’s stress pattern may result from the interplay of several linguistic constraints that potentially influence language production (Calhoun, 2010). These include a tendency for rhythm, but also lexical and syntactic constraints, sentence focus, and information structure, as well as unplanned disfluencies and pauses (Ferreira, 2007). This is supported by studies that induced stress expectations through syntactic (Breen and Clifton, 2011) and information structural predictions (Cutler and Fodor, 1979). In this framework, the position of each stress results from the probabilistic alignment of these constraints with an overall metrical structure (Calhoun, 2010). Prediction at these different levels (e.g., semantic predictions) may therefore contribute to the prediction of stress. And, relatedly, predicting the timing of stressed syllables may be just one of many tools that listeners have for segmentation (Sanders and Neville, 2000) and for allocating attentional resources to new information (Cutler and Fodor, 1979), and may itself be secondary to other mechanisms in everyday speech.

## Conclusion

In this review, we have focused on studies that induced temporal expectations for specific stress patterns based on the idea that stress in speech is rhythmically organized. However, while these studies have often appealed to theories of hierarchical metrical grids, they have typically induced prediction of stress by artificially increasing the amount of periodicity found in speech. While their results are consistent with the notion of neural entrainment involved in musical beat perception, whether this mechanism could account for the prediction of stress in natural speech – presenting far less periodicity – has not been established. We propose that other mechanisms may be at play, such as the prediction of other linguistic features that often coincide with perceived stress.

Nonetheless, the current literature offers insights into the way the prediction of stress through neural entrainment may be utilized for types of speech that naturally present higher degree of rhythmic regularity. For example, infant-directed speech and song present enhanced hierarchical temporal structure than the same materials directed at adults (Falk and Kello, 2017), and child-directed nursery rhymes display hierarchical amplitude modulations that may facilitate the development of phonological awareness through entrainment (Leong and Goswami, 2015). Thus, it is possible that speakers increase the regularity of their own speech to make it easier for listeners to entrain to and predict their stress patterns, consequently rendering speech more intelligible and facilitating language acquisition. Moreover, failure to recruit entrainment mechanisms during development may be fundamentally tied to language deficits such as developmental language disorder and developmental dyslexia (Goswami, 2011). Future studies should aim to establish the degree to which the mechanisms utilized for beat perception in music are applied to different types of speech and in different populations.

## Author Contributions

EB and FF contributed equally to the conceptualization of the arguments and preparation of this manuscript. The initial draft was prepared by EB.

## Conflict of Interest Statement

The authors declare that the research was conducted in the absence of any commercial or financial relationships that could be construed as a potential conflict of interest.
